# Relationship between continuous maternal intrapartum and postpartum glucose monitoring metrics and neonatal outcomes and postpartum glucose metabolism in individuals with gestational diabetes

**DOI:** 10.1186/s12884-026-08944-2

**Published:** 2026-03-16

**Authors:** Karen E. Elkind-Hirsch, Missy Armatta, Edward Veillon, Sara Schiavon, Giacomo Cappon

**Affiliations:** 1 Woman’s Hospital Research Center, Woman’s Hospital, Baton Rouge, LA 70817 USA; 2 Woman’s Diabetes Clinic, Woman’s Hospital, Baton Rouge, LA 70817 USA; 3 Maternal-Fetal Department, Woman’s Hospital, Baton Rouge, LA 70817 USA; 4 Department of Information Engineering (DEI), University of Padova, Padova, 35131 Italy; 5 Dexcom, Inc., Clinical Affairs, 6340 Sequence Drive, San Diego, CA 92121 USA

**Keywords:** Continuous glucose monitoring, Intrapartum glucose management, Gestational diabetes mellitus, Oral glucose tolerance test, Neonatal hypoglycemia, Postpartum glucose metabolism

## Abstract

**Background:**

Continuous glucose monitoring (CGM) improves maternal and neonatal outcomes in pregnancies complicated by preconception diabetes. CGM measurements may help optimize intrapartum and postpartum glycemic management in pregnant women with GDM. We investigated the use of blinded CGM-derived metrics during labor, delivery, and postpartum supplementary to normally monitored glucose measures in women with GDM to study neonatal outcomes and postpartum glucose metabolism.

**Methods:**

This prospective observational study evaluated 60 pregnant women with gestational diabetes mellitus (GDM) during the peripartum and postpartum periods. Of these, 35% were managed with diet alone, while 65% required pharmacologic therapy (insulin, metformin, or both). Participants wore a blinded continuous glucose monitor (Dexcom G6 Pro) for 10 days, beginning 1–3 days before delivery. At 6–18 weeks postpartum, all underwent a 75-g oral glucose tolerance test (OGTT), with a subset (*n* = 28) also using blinded CGM. Univariate and logistic regression analyses assessed associations between CGM metrics in the 24 h preceding delivery and neonatal hypoglycemia or NICU admission.

**Results:**

Infants of women having an induced vaginal or unplanned caesarean delivery (C-section) were significantly more likely than infants delivered by repeat C-section to have neonatal hypoglycemia (*p* = 0.005). No significant correlations between maternal CGM-derived metrics and postnatal adaptation in the newborn were found and none of the models trained to predict neonatal hypoglycemia or intensive unit (NICU) admission showed satisfactory power. While only modest associations between postdelivery CGM metrics and postpartum OGTT values were observed, there were strong correlations between postpartum CGM metrics and OGTT-derived values.

**Conclusion:**

In GDM, intrapartum CGM-based metrics were not associated with adverse neonatal outcomes. Future investigations with a larger cohort are needed to assess intra-and postpartum CGM-metrics in women with GDM and their association with neonatal outcomes and OGTT results.

**Trial registration:**

ClinicalTrials.gov, NCT05067075 (initiation 11/1/2021).

## Background

Gestational diabetes mellitus (GDM) accounts for approximately 86% of all cases of diabetes in pregnancy and, if not well managed, is associated with suboptimal outcomes such as large for gestational age (LGA) infants and neonatal hypoglycemia [1]. Maternal hyperglycemia in women with pregestational or gestational diabetes can cause hypoglycemia in the neonate following delivery [2]. Tight glycemic management during labor and delivery may minimize the risk of neonatal hypoglycemia in pregnant individuals with diabetes [3, 4]. A systematic review examining the relationship between intrapartum maternal glycemic management and neonatal hypoglycemia showed marked heterogeneity in the published studies, with about half showing no association between intrapartum maternal glucose and neonatal hypoglycemia [5].

Use of continuous glucose monitoring (CGM) has been associated with better glycemic management in the third trimester, lower birthweight, and reduced risk of macrosomia in pregnancies complicated by pregestational type 1 diabetes [6]. A few groups have reported on the use of CGM during labor in small pilot studies. Stenninger et al. [7] reported that elevated maternal glucose levels in the last 2 h before delivery correlated with the need for intravenous glucose in the newborn. Cordua et al. [8] examined the effects of CGM on maternal glucose levels in insulin-treated women during labor and delivery, and found lower rates of neonatal hypoglycemia in the CGM arm than in the non-CGM arm. More clinical studies are needed to clarify the intrapartum glycemic profile data in GDM during labor and delivery and its impact on reducing hypoglycemia.

GDM confers an 8-fold increased risk of developing subsequent type 2 diabetes (T2D) [9], so postpartum blood glucose screening is important [10]. While the 75-gram oral glucose tolerance test (OGTT) is the gold standard for postpartum glucose evaluation, it is a cumbersome test that requires fasting, glucose ingestion, and a minimum of 2 h at a healthcare facility, thus screening rates remain low despite these recommendations [10]. Glycemic variability parameters, which could be calculated from CGM data [11] may differ in the progression from the normal to overt diabetes in postpartum women with GDM. Further studies are needed to determine whether appropriate postpartum assessment may be improved by using CGM.

The objective of the current study was to evaluate whether data collected using blinded CGM were useful for predicting neonatal hypoglycemia, NICU admission, and postpartum glucose metabolism in pregnancies complicated by GDM.

## Methods

### Subjects

This prospective observational monocentric study was conducted on pregnant individuals with GDM prior to scheduled delivery at the Woman’s Hospital of Baton Rouge from January 2022 to March 2023. Inclusion criteria included a singleton pregnancy, ≥ 18 years of age, diagnosed with GDM by a 75- or 100-g OGTT, seen in the diabetes clinic and enrolled at their last visit prior to their scheduled delivery date. Participants were excluded if they had pre-existing diabetes, systemic infection, significant comorbidities, prior bariatric surgery, prolonged steroid use, multiple gestation, pre-study glucose-lowering therapy, fetal growth restriction or anomalies, severe psychiatric history, or inability to comply with the protocol. A blinded CGM (Dexcom G6 Pro; Dexcom Inc., San Diego, CA) was placed on subjects for 10 days starting 1–3 days prior to scheduled delivery and returned to the investigators to be downloaded. Labor was routinely induced or caesarean section (C-section) performed between 37 and 40 weeks of gestation. During peripartum and delivery, women were managed per standard of care protocols. Glucose measurements were performed by the point-of-care glucometer available in the delivery room. Glucose levels in diet-only managed patient were monitored once every 4–6 h while glucose was tested every 1–2 h in patients on insulin with levels maintained between 70 and 120 mg/dL (4-6.7 mmol/L). Infants born to mothers with GDM had their blood glucose assessed within 1–2 h of birth. Monitoring frequency was determined by glucose levels: every 1–2 h for low values (< 25–47 mg/dL) and every 2–3 h for borderline values (approximately 47 mg/dL). Glucose was rechecked within one hour after each feeding. Monitoring was discontinued after 12 h if glucose levels remained stable and above 47 mg/dL. Infants who exhibited signs of hypoglycemia received immediate testing.

Participants returned at 6–18 weeks postpartum to be assessed with a standard 75-g OGTT. A subset of participants wore the CGM system for 10 days around the time of their scheduled OGTT. This study was approved by the Institutional Review Board of Woman’s Hospital Foundation.

### Procedures

Clinical and metabolic features of women during pregnancy were obtained from the electronic medical records. The delivery and neonatal outcome data were extracted from the maternal and neonatal birth records and the computerized discharge summary records. All laboratory data were retrieved from the computerized hospital information system. The maternal characteristics included age, race, body mass index (BMI), hypertension (preexisting or pregnancy-induced), family history of diabetes, and previous GDM. Delivery methods were categorized as primary C-section, elective (repeat) C-section, or induced vaginal delivery. Neonatal hypoglycemia (NH) was defined as one or more episodes of consecutive blood glucose concentrations of < 47 mg/dL (< 2.6 mmol/L) within the first 48 h after delivery. Large and small for gestational age were defined by 90th (LGA) and 10th (small-for gestational age) percentiles for our population, respectively. Infant variables including hypoglycemia, neonatal intensive care unit (NICU) admission, and Apgar scores were obtained from medical records.

OGTTs were performed at 6–18 weeks postpartum after an overnight fast. After the collection of a baseline blood sample, a 75-g oral glucose load was administered; additional blood samples were drawn 30, 60, and 120 min later into potassium oxalate tubes for the measurement of glucose and lithium heparin tubes for insulin levels and to assess glycemic status, fasting (FBG) and mean blood glucose (MBG), insulin resistance and pancreatic beta-cell function. Glucose tolerance was defined as normal, impaired (impaired fasting glucose (IFG), impaired glucose tolerance (IGT) or IFG/IGT) or diabetic according published criteria [12]. Glucose concentrations were measured using the Vitros Chemistry System (Ortho Vitros 5600 System; Raritan, NJ) and insulin levels were analyzed using a Beckman Coulter Access 2 Analyzer (Beckman Coulter, Brea, CA).

### Calculations

Mean OGTT glucose was defined as the average value of glucose measures during the OGTT and calculated by summing glucose values obtained and dividing by 4. Insulin sensitivity was calculated by using the insulin sensitivity index (OGTT ISI) of Matsuda and DeFronzo [13]. Insulin secretion was estimated by the insulinogenic index (IGI) calculated from the ratio of increments of serum insulin to glucose measured at 30 min [14]. Integrated β cell function was measured by the oral disposition index (oDI) as the product of insulin secretion and insulin sensitivity [15].

### CGM Data

Extraction of CGM-derived features was performed using the Automated Glucose dATa Analysis (AGATA) toolbox [16] with the pregnancy-specific target range of 63–140 mg/dL (3.5–7.8 mmol/L) following the definitions recommended by an international consensus on CGM-derived metrics for clinical trials involving pregnant women [17]. CGM-derived metrics were computed only when sensor data wear time exceeded 70% of the monitoring period. Specifically, the following CGM variables were included in the analyses: the standard deviation of glucose (SD), coefficient of variation for glucose (%CV), time in range (TIR), time in tight range (TITR), time in hyperglycemia, time in hypoglycemia, glucose time management indicator (GMI) and mean amplitude of glycemic excursions (MAGE). The %CV was calculated by dividing the glucose standard deviation by the mean glucose. The GMI indicates the A1C level that would be expected based on glucose levels from CGM readings over 10–14 days. The MAGE which was designed to quantify major swings of glycemia, was used for assessing intra-day glycemic variability in this study.

### Predictive models of NH and NICU admission using CGM data

Univariate correlation analysis was initially performed to examine the relationship between CGM-derived glucose metrics and the two main neonatal clinical outcomes of neonatal hypoglycemia (NH) and neonatal Intensive care unit (NICU) admission.

Logistic regression models were further used to investigate the relationship between CGM data in the 24 h before delivery and neonatal outcomes of NH and NICU admission.

The features derived from the CGM data prior to delivery were used together with maternal characteristics to train linear predictive models to be used to predict, prior to delivery, NH and/or admission to the NICU. Linear models were selected due to the limited sample size in the dataset. More complex machine learning approaches were avoided to prevent overfitting, as established in the literature for datasets with only 60 observations. After defining the datasets, we utilized both linear logistic regression (LL) and least absolute shrinkage and selection operator (LASSO) logistic regression models to predict each neonatal outcome. Dataset A included maternal variables (age, delivery method, medication history) and newborn variables (sex, weight, length, gestational age). Dataset B incorporated all Dataset A features plus continuous glucose monitoring (CGM) metrics from the 24 h prior to delivery, such as average glucose, standard deviation, coefficient of variation, mean amplitude of glycemic excursions (MAGE), and the percentage of time spent in specific glycemic ranges: level 1 hypoglycemia (54–63 mg/dL), level 2 hypoglycemia (< 54 mg/dL), level 1 hyperglycemia (140–250 mg/dL), level 2 hyperglycemia (> 250 mg/dL), and the target range (63–140 mg/dL). Dataset C included all Dataset A variables and CGM metrics from Dataset B, calculated over the entire 10-day CGM period. Subjects with less than 20 h of CGM data or less than 90% data completeness were excluded. Training was performed in a standard stratified split 5-fold cross-validation setup. This particular scheme was adopted to ensure an equal distribution of the classes of the neonatal outcomes to be predicted in each fold used for model training and testing. In addition, numerical variables were normalized using the “min-max scaling” technique, ${x}_{norm}=\left(\frac{x-\mathrm{min}\left(x\right)}{\mathrm{max}\left(x\right)-\mathrm{min}\left(x\right)}\right),$ to ensure that they had the same impact on predicting the considered outcomes. LL and LASSO classification accuracy were quantified in terms of specificity, precision, accuracy, recall, and F1-score.

### Statistical analysis

Based on prior studies showing a correlation (*r* = 0.42) between maternal glucose and neonatal hypoglycemia, a sample size of 60 was calculated to achieve 80% power at a two-sided 5% significance level, allowing for a 15% dropout rate. Data analysis was performed using the IBM SPSSV. 29.0 statistical analysis program (IBM Corp., Armonk, NY) with *p* < 0.05 from the two-side test considered significant. Continuous variables were expressed as mean ± standard deviation (SD); categorical variables were summarized as counts and percentages. Spearman correlation analysis was used to further explore the relationships between CGM metrics and neonatal hypoglycemia and NICU admission. The clinical data and other variables were compared using one-way ANOVAs for continuous variables. The composition ratio (%) of count data was described, and a chi-square (χ2 test) or Fisher’s exact probability method was used for categorical variables. Pearson and point biserial correlation analyses were performed to examine the relationship between of CGM metrics and OGTT-derived glucose and insulin values as well as maternal and neonatal clinical measures.

## Results

Sixty women with GDM (41 White, 17 Black, 1 Asian, 1 Middle Eastern) were evaluated. The mean (SD) age was 30.9 (5.6) years (range, 19–42 years), mean BMI was 39.5 (9.1) kg/m^2^ (range, 20.5–63.4 kg/m^2^), and mean gestational age was 38.3 (1.03) weeks (range, 36–40 weeks). Forty-five (75%) women received a GDM diagnosis prior to 26 weeks, 47 (78%) of the women had a family history of diabetes, and 24 (40%) had hypertension. Of the 38 multigravid women, 21 (55%) been diagnosed with GDM in a previous pregnancy. Twenty-four women were treated with diet alone, six with diet and metformin, 19 with diet and insulin, and 14 with diet, metformin, and insulin. Thirty-nine (65%) of the 60 neonates experienced hypoglycemia. As shown in Table 1, the rate of hypoglycemia among infants delivered via elective C-Sect.  (4/14, 28.6%) was significantly lower than that for those delivered by either primary C-Sect.  (10/13, 76.9%) or vaginally (25/33, 75.8%) (χ^2^ = 10.7; *p* < 0.01).

**Table 1:** Presence of neonatal hypoglycemia by sub-types of delivery method

Delivery Method	Neonatal hypoglycemia	Absence of neonatal hypoglycemia	Total
Vaginal delivery	25	8	33
Elective C-section	4	10	14
Primary C section	10	3	13
Total	39	21	60

χ²test = 10.7, *P* = 0.005

Neonates experiencing or not experiencing hypoglycemia were similar with respect to Apgar scores and birth weights, but those with hypoglycemia were more likely to be admitted to the NICU. Infants born to women managed with pharmacotherapy had rates of LGA (9/39, 23%) and hypoglycemia (28/39, 72%) that were significantly higher than rates for infants born to women managed with diet alone (11/21, 52% for hypoglycemia and 0/21 for LGA). There were no differences in Apgar scores in neonates of mothers treated with diet or medication.

As shown in Table 2, univariate analyses did not demonstrate any significant correlations between CGM-derived metrics from the 24 h before delivery and rates of neonatal hypoglycemia or NICU admission.

**Table 2:** Correlation of CGM Metrics 24 h Before Delivery with Hypoglycemia or NICU Admission. CV, Coefficient of variation; SD, Standard deviation; MAGE, Mean amplitude of glycemic excursion, R, Spearman rank correlation coefficient

CGM Metric	Mean	SD	Neonatal Hypoglycemia		NICU Admission	
			*R*	*p* value	*R*	*p* value
Mean Glucose (mg/dL)	105.7	21.3	-0.24	0.09	-0.047	0.25
Time in Target Range (63–140 mg/dL)	86,3	17.2	0.076	0.7	0.15	0.10
Time in Tight Target (63–120 mg/dL)	83.4	17.4	0.016	0.41	0.10	0.45
CV of Glucose (%)	18.3	5.6	-0.17	0.12	-0.058	0.30
SD of Glucose (mg/dL)	19.6	8.9	-0.256	0.08	-0.057	0.30
MAGE( mg/dL)	36.2	15.8	-0.054	0.29	-0.053	0.26
Time in Hyperglycemia (> 140 mg/dL)	11.2	17.3	0.097	0.95	-0.20	0.097
Time in Hypoglycemia (< 63 mg/dL)	2.5	6.8	0.056	0.3	0.11	0.68

As shown in Table 3, more complex strategies were employed to investigate the relationship between CGM data in the 24 h before delivery and neonatal outcomes. We first trained the model using only maternal and newborn features (Dataset A). Incorporation of blinded CGM measures from the 24 h close to delivery (Dataset B) did not seem to improve the predictive power of the models. Predictions obtained from Dataset C (maternal and newborn attributes and CGM metrics from ~ 10 days surrounding delivery) were slightly better than those performed considering only the 24 h before delivery, but they were not significantly better than those obtained considering only the maternal and neonatal outcome dataset.

**Table 3:** Specificity, precision, accuracy, recall, and F1-scores for two different models across three datasets for the outcomes of neonatal hypoglycemia and NICU admission. LL, Logistic classification model; LASSO, Least absolute shrinkage and selection operator model; NaN, Not a number. Dataset A: maternal and newborn features only; Dataset B: Integration of CGM-based metrics from the 24 h prior to delivery; Dataset C: Integration of CGM-based metrics from the 10 days surrounding delivery.

Dataset	Model	Specificity	Precision	Accuracy	Recall	F1-Score
Outcome: Neonatal Hypoglycemia						
A	LL	0.673	0.714	0.633	0.600	0.627
	LASSO	0.666	0.735	0.700	0.729	0.728
B	LL	0.640	0.696	0.625	0.610	0.647
	LASSO	0.520	0.665	0.625	0.705	0.677
C	LL	0.547	0.617	0.567	0.571	0.585
	LASSO	0.547	0.655	0.633	0.690	0.669
Outcome: NICU Admission						
A	LL	0.609	0.194	0.567	0.367	NaN
	LASSO	0.933	NaN	0.733	0.0	NaN
B	LL	0.544	0.073	0.471	0.200	NaN
	LASSO	0.953	NaN	0.776	0.100	NaN
C	LL	0.698	0.180	0.617	0.300	NaN
	LASSO	0.933	NaN	0.750	0.067	NaN

Forty-six of the 60 participants (77%) returned for postpartum glucose testing. Three participants were diagnosed with T2D (fasting glucose > 125 mg/dL) and did not complete the postpartum OGTT. Of the participants studied, 44% had impaired glucose metabolism postpartum; IFG (12.2%), IGT (12.2%), IFG/IGT (14.6%) or T2D (5%). Table 4 presents correlations between CGM-based and OGTT-based metrics within 1 week of delivery or at 6–18 weeks post-delivery. Mean OGTT glucose concentrations were weakly but significantly related to mean glucose and glycemic variability (SD and %coefficient of variation (CV)) at 1 week of delivery and inversely with time in tight range. The oDI was slightly but significantly inversely correlated to indices of glycemic variability. In contrast, mean OGTT glucose concentrations correlated significantly with mean glucose and glycemic variability (SD and %CV) at 6–18 weeks postpartum and inversely with time in tight range. The oDI was significantly inversely correlated to indices of glycemic variability and was positively correlated with TITR at 6–18 weeks postpartum.

**Table 4:** Correlation of CGM metrics with postpartum OGTT indices. OGTT, Oral glucose tolerance test; SIOGTT, Insulin sensitivity index; IGI, Insulinogenic index; oDI, oral disposition index; TITR, Time in tight range (63–140 mg/dL); CV, Coefficient of variation

Interval	CGM Metrics	OGTT Metrics			
		Mean Glucose	SI_OGTT_	IGI	oDI
Postdelivery 1 to 7 Days (*n* = 41)	Mean glucose	*r* = 0.32	*r* = -0.35	*r* = -0.03	*r* = -0.10
		*p* < 0.04	*p* < 0.025	p = NS	p = NS
	GMI	*r* = 0.32	*r* = -0.35	*r* = -0.03	*r* = -0.10
		*p* < 0.04	*p* < 0.025	p = NS	p = NS
	TITR (%)	*r* = -0.32	*r* = 0.29	*r* = 0.09	*r* = 0.2
		*p* < 0.04	p = NS	p = NS	p = NS
	SD (mean)	*r* = 0.47	*r* = -0.28	*r* = -0.21	*r* = -0.33
		*p* < 0.002	p = NS	p = NS	*p* < 0.035
	CV (%)	*r* = 0.35	*r*= -0.11	*r*= -0.29	*r*= -0.35
		*p* < 0.02	p = NS	p = NS	*p* < 0.024
Postpartum 6 to 18 weeks (*n* = 21)	Mean glucose	*r* = 0.72	*r*= -0.43	*r* = -0.24	*r*= -0.48
		*p* < 0.001	*p* < 0.023	p = NS	*p* < 0.009
	GMI	*r* = 0.72	*r*= -0.43	*r* = -0.24	*r*= -0.48
		*p* < 0.001	*p* < 0.023	p = NS	*p* < 0.009
	TITR (%)	*r* = -0.74	*r* = 0.35	*r* = 0.31	*r* = 0.47
		*p* < 0.001	p = NS	p = NS	*p* < 0.01
	SD (mean)	*r* = 0.85	*r* = -0.28	*r* = 0.43	*r* = -0.55
		*p* < 0.0001	p = NS	*p* < 0.021	*p* < 0.003
	CV (%)	*r* = 0.64	*r* = -0.05	*r* =-0.48	*r*=-0.45
		*p* < 0.001	p = NS	*p* < 0.01	*p* < 0.015

## Discussion

In women with type 1 diabetes and type 2 on insulin and women with diabetes treated with antenatal corticosteroids, maternal hyperglycemia in 24-hour prior to birth is associated with neonatal hypoglycemia [2, 3]. Stenninger et al. [7] reported a direct association between maternal glucose concentrations using a CGM in mothers with insulin-treated diabetes during the 24 h prior to delivery and neonatal hypoglycemia. They found that multiple glycemic indices correlated with the need for neonatal intravenous glucose infusions.

In the current study, we did not find that the blinded CGM-derived metrics of glycemia or variability 24 h before delivery were correlated with hypoglycemia in the newborn in women with GDM, nor did the integration of CGM data with maternal characteristics improve the predictive performance of the adopted regression models for postnatal glucose adaptation and neonatal hypoglycemia. The observation that glucose metrics of the mother and neonatal hypoglycemia were not related was unanticipated. Determining an operational threshold for neonatal hypoglycemia remains a significant clinical challenge. In this study, hypoglycemia was defined as a blood glucose concentration of < 47 mg/dL (< 2.6 mmol/L) in infants born to mothers with diabetes or gestational diabetes, with 39 of 60 infants (65%) meeting this criterion. Using a slightly lower threshold of 45 mg/dL (< 2.5 mmol/L), Cordua et al. [8] reported hypoglycemia in 37% of infants with mothers monitored with real-time continuous glucose during labor, compared with 46% in the control group. Stenninger et al. [7] identified hypoglycemia in 60% of infants of diabetic mothers using a threshold of 39.6 mg/dL (< 2.2 mmol/L), with 33% requiring intravenous glucose therapy. Our less stringent definition of hypoglycemia may have contributed to the absence of an observed association between blinded intrapartum CGM metrics and neonatal hypoglycemia.

However, while some studies have reported neonatal hypoglycemia to be associated with maternal glucose management during delivery, a recent review found that the majority of studies were discordant in finding an association between intrapartum maternal glucose and neonatal hypoglycemia [5] and factors occurring well before parturition pose a more substantial risk for neonatal hypoglycemia than intrapartum maternal glycemic management.

Glucose and insulin requirements have been demonstrated to vary during labor and delivery in pregnancies complicated by diabetes [18]. We observed that mode of delivery had a significant impact on neonatal hypoglycemia with infants of mothers having either a primary C- section or induction, thus having a longer time in active labor, significantly more likely than women having a repeat C-section to have newborns with neonatal hypoglycemia. Whether restrictive glucose infusion or higher insulin doses during labor and delivery, without the expense of maternal hypoglycemia, may reduce the prevalence of neonatal hypoglycemia is yet to be evaluated. While there were no differences in Apgar scores between hypoglycemic neonates and euglycemic neonates, those with hypoglycemia were more likely to be admitted to the NICU. Glycemic management during pregnancy has also been associated with neonatal hypoglycemia.

The complications of GDM may indeed be greater based on the severity of maternal glycemia. Kole et al. [19] found that medical management compared with dietary management was associated with an increased risk of neonatal hypoglycemia. In agreement, we found neonatal hypoglycemia occurring in 52% of pregnancies managed with diet al.one compared to 72% in those managed with metformin, insulin or combination. We also observed that mothers treated medically during pregnancy had a greater rate of LGA neonates compared to diet treatment. The incidences of neonatal hypoglycemia were significantly higher in infants born LGA. Maternal hyperglycemia has long been considered the principal determinant of LGA and the factor most amenable to intervention [20].

Women who have had GDM are at increased risk for developing varying impaired glucose tolerance after delivery [9, 10]. In this study, we found that 44% of the women who underwent OGTT testing had impaired glucose metabolism. Nevertheless, despite the current guidelines indicating a postpartum OGTT in women with previous GDM, the adherence rate to this recommendation remains unsatisfactory being widely below 50% [21, 22]. Even is this trial, only 77% of our participants returned for postpartum testing.

Few investigations have described glycemic profiles in the early time period after delivery and how glucose abnormalities on CGM relate to OGTT value. Recently, Cabrera et al. [23] assessed the use of blinded CGM for the detection of postpartum dysglycemia and compared its results to standard of care OGTT in people with recent GDM. First blinded sensors were placed on postpartum day 1–3 before discharge from the hospital They found that placement of CGM at discharge was a convenient initial postpartum screen for postpartum dysglycemia with high completion rates, sensitivity, and acceptability ratings [23]. In assessing the immediate post-delivery (+ 1–7 days) CGM-metrics, we found there was a positive correlation of measures of glycemia and variability and negative association of time-in-tight range (TITR) with the postpartum mean OGTT glucose values. The oral disposition index (oDI) was negatively related to CGM indices of variability but not with glycemia. Furthermore, the second 6-24-week postpartum sensor showed strong associations with OGTT-derived indices and postpartum CGM-metrics. Mean OGTT glucose concentrations correlated significantly with mean glucose and glycemic variability and inversely with TITR. While OGTT ISI was slightly inversely correlated with MBG and GMI but not to glycemic variability, the IGI, a surrogate marker of early insulin response, was significantly negatively related to glycemic variability while the oDI was significantly inversely correlated with glycemic variability and glycemia and positively correlated with TITR. The results of our study are consistent with those in nondiabetic and newly-diagnosed subjects with T2D [11]. Increased glycemic variability parameters were reported to be consistently associated with decreased oDI in subjects across the range of glucose tolerance from normal glucose regulation to impaired glucose metabolism to diabetes by isolated 2-h glucose to T2D group [11]. Wang et al. [24] showed that patients with diabetes monitored by a CGM had increased postprandial glucose excursion, higher glucose levels overnight and greater inter-day fluctuations compared with the normoglycemic and impaired glucose regulation individuals. Using OGTT results as the standard, we found that blinded CGM conducted concurrently demonstrated a significant correlation with postpartum dysglycemia detection in individuals with recent GDM. Future research should involve large, multicenter studies to establish optimal glucose thresholds and timing for CGM and should compare these findings to the true gold standard—future diabetes risk. Additionally, it is important to further evaluate whether postpartum CGM can serve as a diagnostic tool or as a trigger for performing an OGTT.

The strengths of this study are its ethnically and economically diverse population. The small sample size represents a limitation to generalize our findings. It is further limited by the observational nature of the blinded data. The data presented here represent a single center’s experience. The heterogeneity of delivery method - elective C-section versus induced delivery - may have impacted the findings given that labor is associated with an increase in both glucose utilization and production. Subsequent research should include a large, multicenter study to establish of the relationship among real-time CGM metrics during labor and delivery in women with GDM and neonatal hypoglycemia and NICU admission.

## Conclusions

This is the first report looking at whether the use of blinded glucose monitoring during labor, delivery, and early postpartum in women with GDM would provide useful information to improve glycemia during labor in this diabetic population. The integration of CGM data with maternal characteristics did not prove effective in enhancing the predictive performance of the adopted regression models for postnatal glucose adaptation and neonatal hypoglycemia. While there was no direct relationship, it suggests that intrapartum glycemic management may be less important than the overall glucose management during pregnancy. Future clinical trials should include in their design rt CGM technology during labor and impact of glucose management on neonatal outcomes. As a screening tool, CGM has potential advantages and limitations when compared to the OGTT. Future studies with a larger cohort are needed to assess CGM- metrics postpartum and their relationship with postpartum OGTT results for detecting early dysglycemia in the postpartum GDM population. Whether postpartum CGM can be used as a diagnosis or initiate the need to have an OGTT performed needs to be more thoroughly evaluated.

## Data Availability

Some or all datasets generated during and/or analyzed during the current study are not publicly available but are available from the corresponding author on reasonable request.

## Abbreviations

CGM: Continuous glucose monitoring
GDM: Gestational diabetes
OGTT: Oral glucose tolerance tests
NICU: Neonatal intensive care unit
LGA: Large for gestational age
T2D: Type 2 diabetes
C-section: Caesarean section
BMI: Body mass index
NH: Neonatal hypoglycemia
FBG: Fasting blood glucose
MBG: Mean blood glucose
IFG: Impaired fasting glucose
IGT: Impaired glucose tolerance
ISI: Insulin sensitivity index
IGI: Insulinogenic index
oDI: Oral disposition index
AGATA: Automated Glucose dATa Analysis
GMI: Glucose management indicator
HbA1c: Glycated hemoglobin
LL: Linear logistic classification model
LASSO: Least absolute shrinkage and selection operator
SD: Standard deviation
CV: Coefficient of variation
TITR: Time-in-tight range
